# Supplementary material for: Phenome-wide association study of loci harboring de novo tandem repeat mutations in UK Biobank exomes
Source: Nat Commun. 2022 Dec 12;13:7682. doi: 10.1038/s41467-022-35423-x (PMC9744822; doi:10.1038/s41467-022-35423-x)
Supplement: Supplementary file 1 — Supplementary Information [file 41467_2022_35423_MOESM1_ESM.pdf]

SUPPLEMENTARY FIGURES

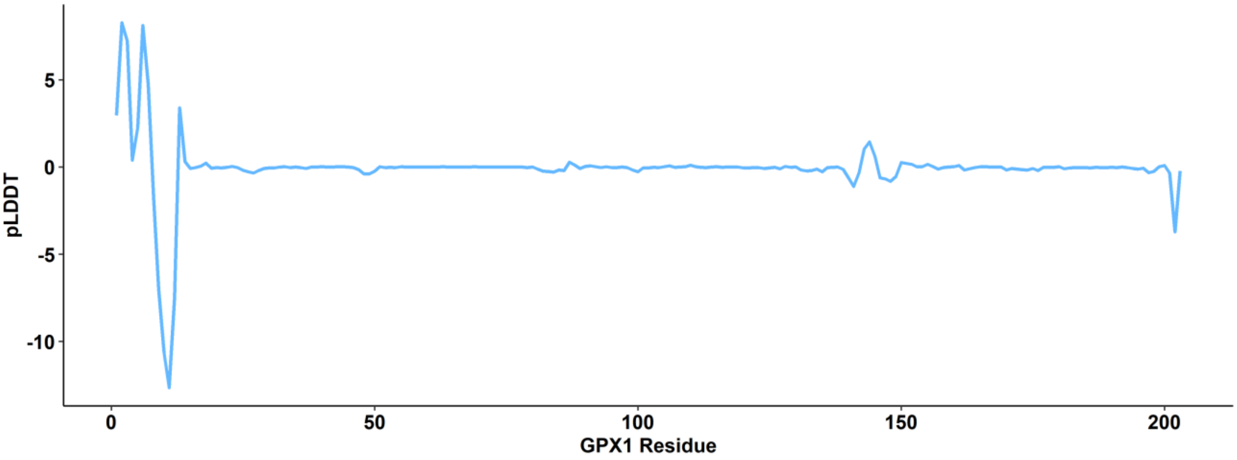

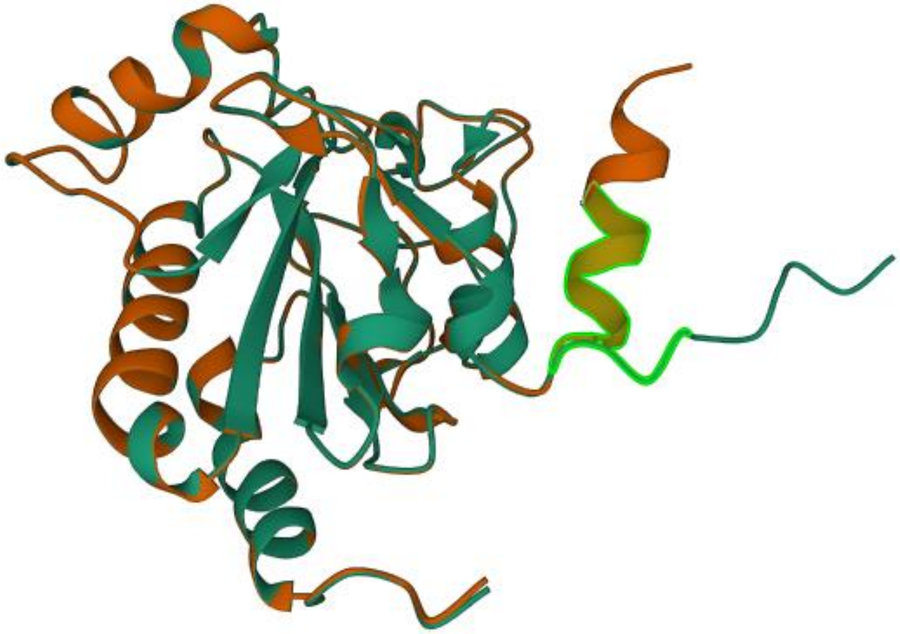

Figure S1. Putative impact of tandem repetitive mutations on the structure of GPX1. The per-residue folding confidence in canonical *GPX1* (*GPX1*-$[GCC]_7$) was compared to the short mutation (*GPX1*-$[GCC]_4$). Mol* Viewer was used to superpose the predicted structures with the GCC-containing stretch of alanines highlighted in the green structure (short) and orange structure (canonical).

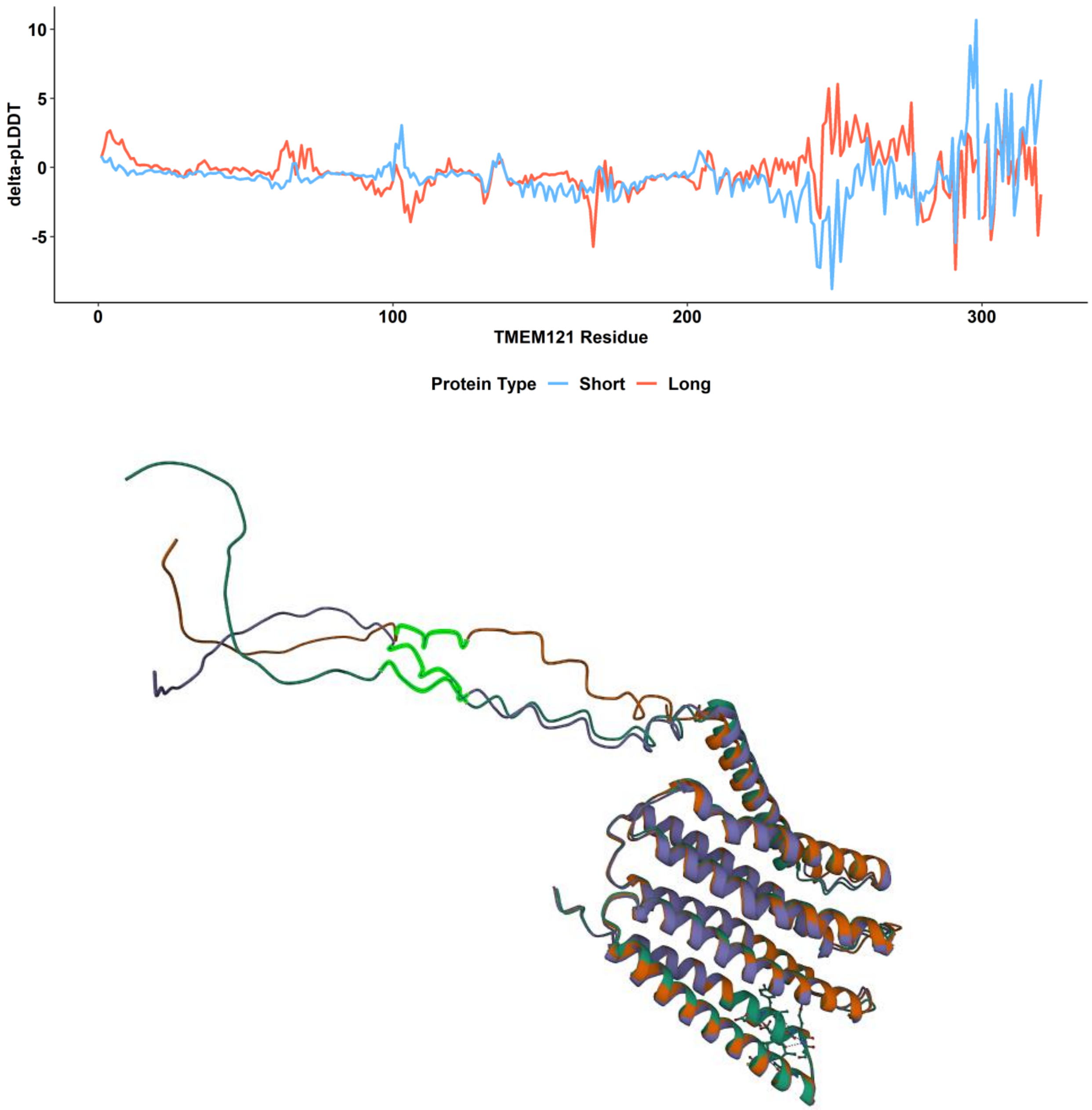

Figure S2. Putative impact of tandem repetitive mutations on the structure of TMEM121. The per-residue folding confidence in canonical *TMEM121* (*TMEM121*-[GCC]$_6$) was compared to the short (*TMEM121*-[GCC]$_5$) and long (*TMEM121*-[GCC]$_7$) mutations. Mol* Viewer was used to superpose the predicted structures with the GCC-containing stretch of prolines highlighted in the green structure (short), orange structure (canonical), and purple structure (long).

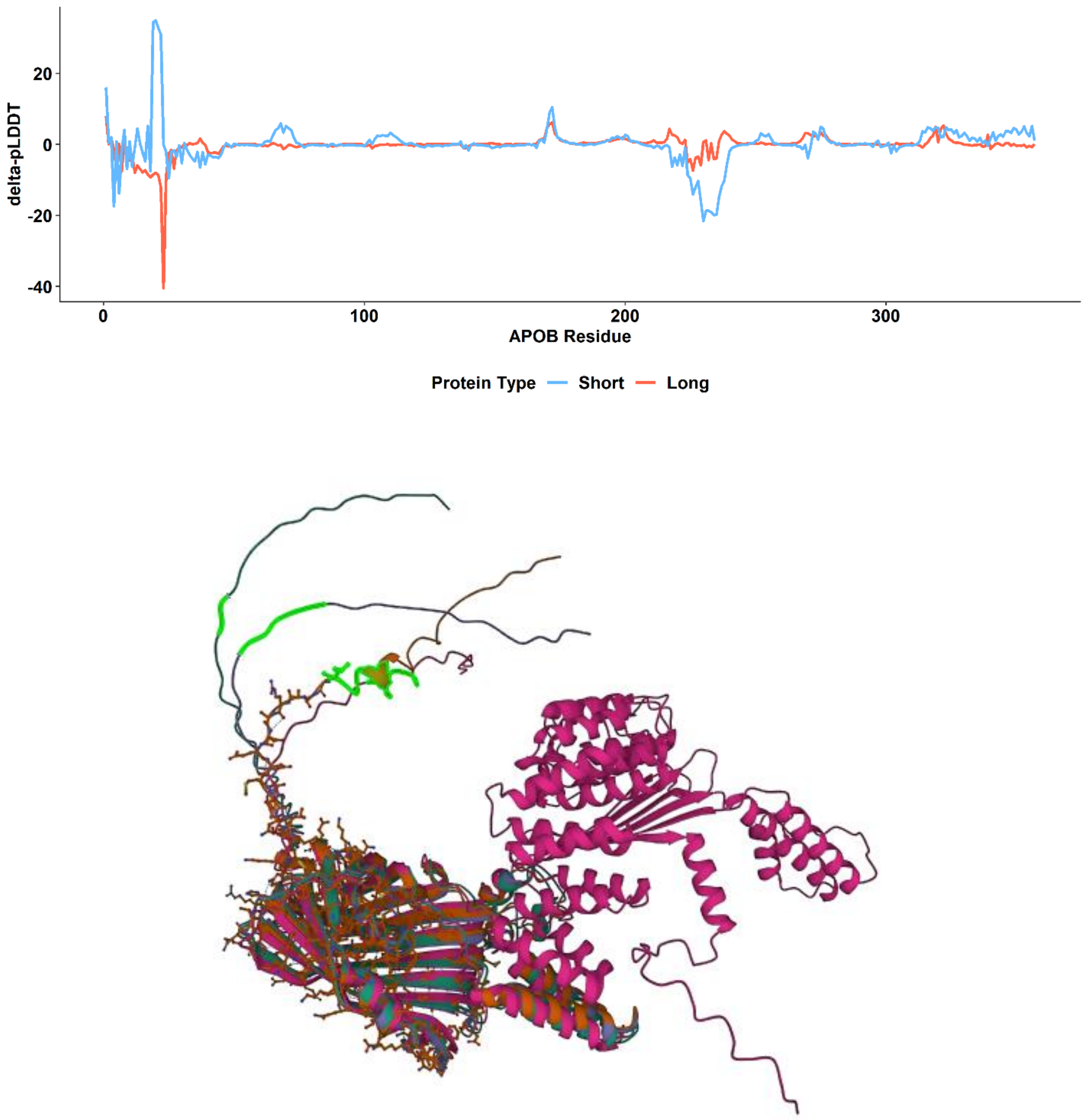

Figure S3. Putative impact of tandem repetitive mutations on the structure of APOB. The per-residue folding confidence in canonical *ABOP* (*ABOP*-[CAG]$_6$) was compared to the short (*ABOP*-[GCC]$_2$) and long (*ABOP*-[CAG]$_7$) mutations. Mol* Viewer was used to superpose the predicted structures. Note that they size of APOB prohibited AlphaFold structure prediction for the entire protein so we predicted the structure of one segment of APOB (see Jumper, et al. 2021, PMID 34265844). The GCC-containing stretch of leucines is highlighted in the green structure (short), orange structure (long), purple structure (canonical sequence at truncated AlphaFold length), and pink structure (full length APOB canonical sequence).

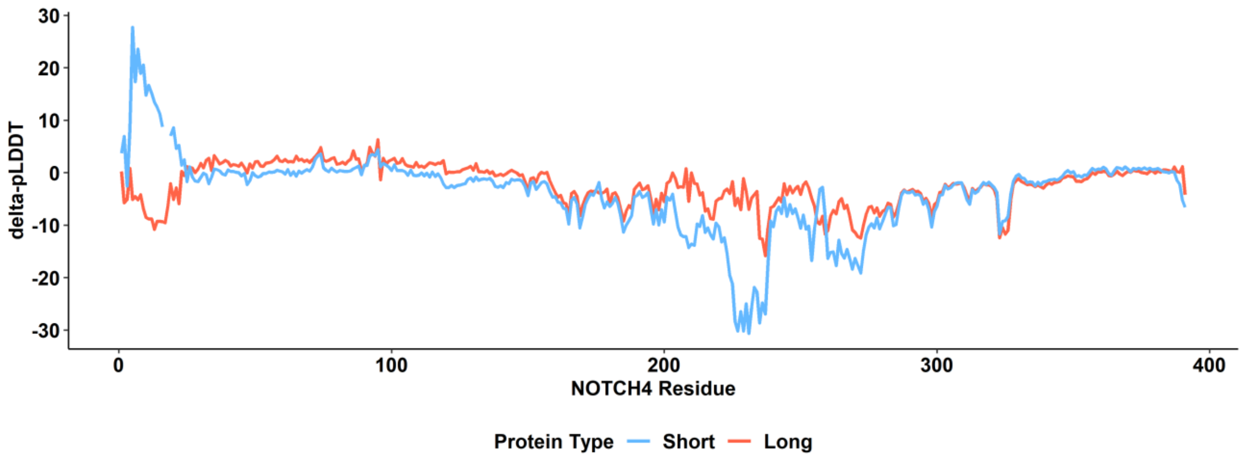

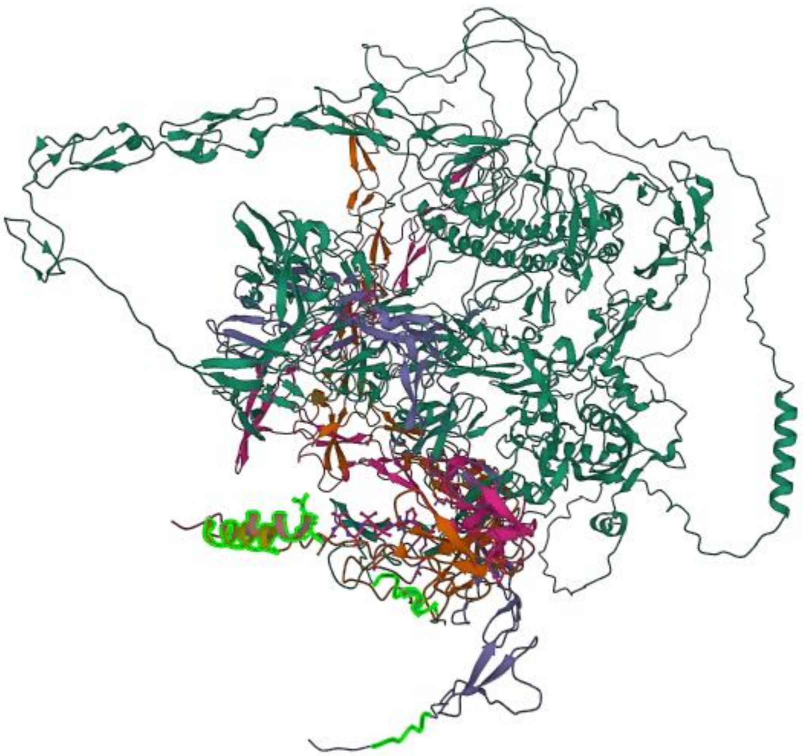

Figure S4. Putative impact of tandem repetitive mutations on the structure of NOTCH4. The per-residue folding confidence in canonical *NOTCH4* (*NOTCH4*-[AGC]$_{11}$) was compared to the short (*NOTCH4*-[AGC]$_{6}$) and long (*NOTCH4*-[AGC]$_{13}$) mutations. Mol* Viewer was used to superpose the predicted structures. Note that they size of NOTCH4 prohibited AlphaFold structure prediction for the entire protein so we predicted the structure of one segment of NOTCH4 (see Jumper, et al. 2021, PMID 34265844). The AGC-containing stretch of leucines is highlighted in the green structure (full length NOTCH4 canonical sequence), orange structure (long), purple structure (short), and pink structure (canonical sequence at truncated AlphaFold length).

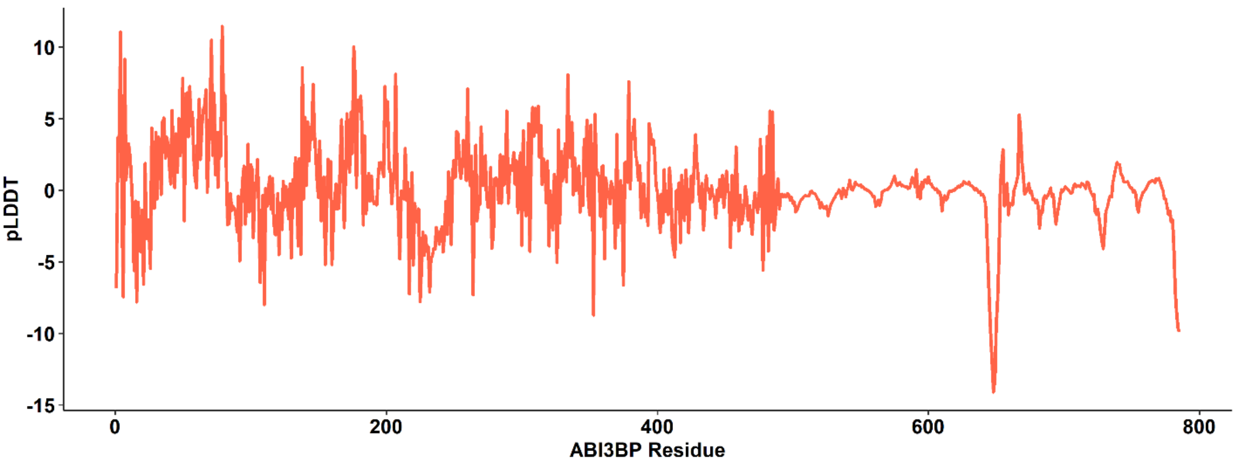

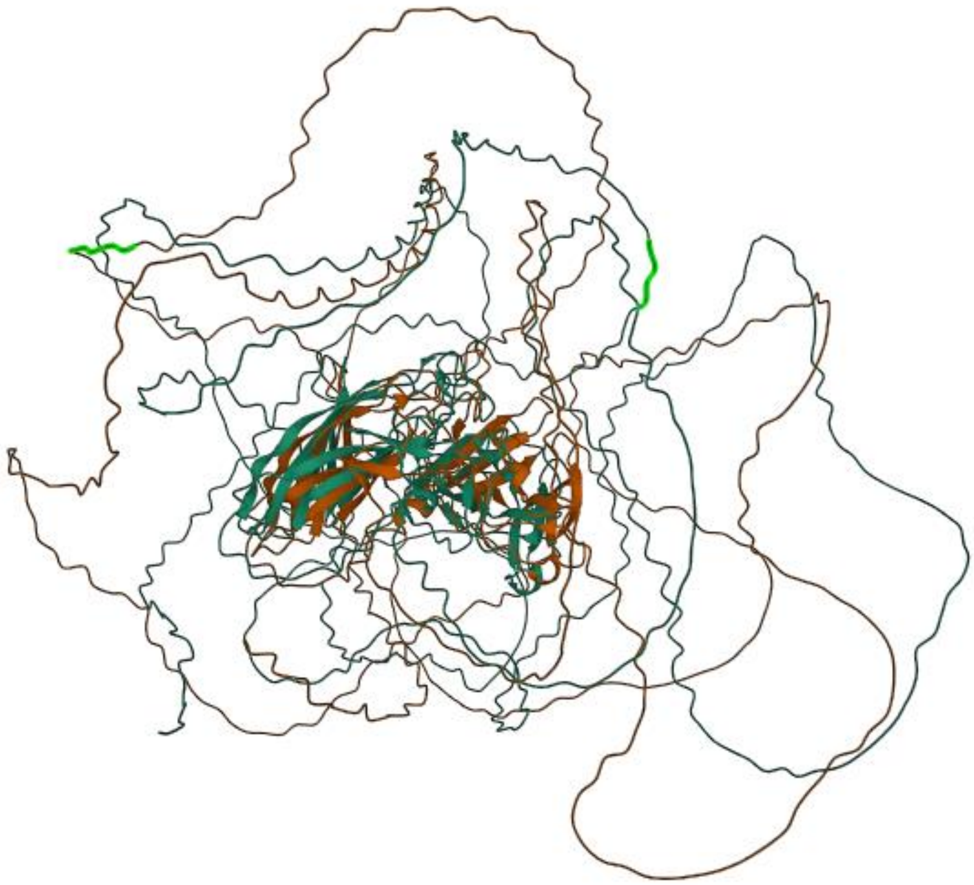

Figure S5. Putative impact of tandem repetitive mutations on the structure of ABI3BP. The per-residue folding confidence in canonical *ABI3BP* (*ABI3BP*-[TTG]$_6$) was compared to the long (*ABI3BP*-[TTG]$_5$) mutation. Mol* Viewer was used to superpose the predicted structures with the TTG-containing stretch of threonines highlighted in the green structure (canonical) and orange structure (long).
