# Supplementary material for: Phenome-wide association study of loci harboring de novo tandem repeat mutations in UK Biobank exomes
Source: Nat Commun. 2022 Dec 12;13:7682. doi: 10.1038/s41467-022-35423-x (PMC9744822; doi:10.1038/s41467-022-35423-x)
Supplement: Supplementary file 3 — Description of Additional Supplementary Files [file 41467_2022_35423_MOESM3_ESM.pdf]

DESCRIPTION OF ADDITIONAL SUPPLEMENTARY FILES

Supplementary Data 1. European family trios. Seven trios had no detectable *de novo* TR mutations based on the quality control thresholds applied in this study.

Supplementary Data 2. All *de novo* TR mutations detected across 39 UKB family trios of European descent.

Supplementary Data 3. Gene-sets enriched for loci harboring de novo TR mutations using ShinyGO and FUMA. Enrichments are calculated using one-sided hypergeometric tests. Gene sets highlighted in yellow are significant after multiple testing correction using both methods.

Supplementary Data 4. Expression-associated TRs across 17 tissue types from GTEx. Empty cells indicate that a TR was not present in the TR-expression study performed by Fotsing, et al.[15] Beta, se, and p-value reflect linear models of gene expression using the indicated TR locus including sex, population structure, and technical covariates.

Supplementary Data 5. All significant associations between TRs and UK Biobank phenotypes. Each row describes a single TR-phenotype association. The UKB Field ID, full trait description, and trait domain are provided.

Supplementary Data 6. Comparison of TR length sum effects for select TR-phenotype associations. Cohen's *d* is reported for each outcome comparing each pair of TR lengths with at least 7 observations. Associated P-values were calculated using two-sided Z-tests.

Supplementary Data 7. Per-locus enrichment of associated trait domains using one-sided hypergeometric tests. Highlighted cells indicate significant enrichment after multiple testing correction (FDR<5%).

Supplementary Data 8. Significant fine-mapping results for TR-phenotype associations from Supplementary Data 5.

Supplementary Data 9. Comparison of TR-trait effect estimates in the population-based PheWAS and in probands from the family-based design phase. Effect estimates reflect linear regression models.

Supplementary Data 10. TRs with high fine-mapping probabilities annotated to protein domains. Green indicates proteins with exonic localization subjected to structure prediction with AlphaFold.

Supplementary Data 11. Comparison of regional alignment error between canonical and mutated FNBP4 based on AlphaFold prediction of protein structure. Differences were tested using two-sided Z-tests.

Supplementary Data 12. Comparison of regional alignment error between canonical and mutated BTN2A1 based on AlphaFold prediction of protein structure. Differences were tested using two-sided Z-tests.
