# Supplementary material for: Phenome-wide association study of loci harboring de novo tandem repeat mutations in UK Biobank exomes
Source: Nat Commun. 2022 Dec 12;13:7682. doi: 10.1038/s41467-022-35423-x (PMC9744822; doi:10.1038/s41467-022-35423-x)
Supplement: Supplementary file 5 — Reporting Summary [file 41467_2022_35423_MOESM5_ESM.pdf]

nature portfolio

Corresponding author(s): Frank Wendt

Last updated by author(s): Nov 19, 2022

Reporting Summary

Nature Portfolio wishes to improve the reproducibility of the work that we publish. This form provides structure for consistency and transparency in reporting. For further information on Nature Portfolio policies, see our Editorial Policies and the Editorial Policy Checklist.

Statistics

For all statistical analyses, confirm that the following items are present in the figure legend, table legend, main text, or Methods section.

n/a	Confirmed	
☐	☒	The exact sample size (*n*) for each experimental group/condition, given as a discrete number and unit of measurement
☒	☐	A statement on whether measurements were taken from distinct samples or whether the same sample was measured repeatedly
☐	☒	The statistical test(s) used AND whether they are one- or two-sided<br>*Only common tests should be described solely by name; describe more complex techniques in the Methods section.*
☐	☒	A description of all covariates tested
☐	☒	A description of any assumptions or corrections, such as tests of normality and adjustment for multiple comparisons
☐	☒	A full description of the statistical parameters including central tendency (e.g. means) or other basic estimates (e.g. regression coefficient) AND variation (e.g. standard deviation) or associated estimates of uncertainty (e.g. confidence intervals)
☐	☒	For null hypothesis testing, the test statistic (e.g. *F*, *t*, *r*) with confidence intervals, effect sizes, degrees of freedom and *P* value noted<br>*Give P values as exact values whenever suitable.*
☒	☐	For Bayesian analysis, information on the choice of priors and Markov chain Monte Carlo settings
☒	☐	For hierarchical and complex designs, identification of the appropriate level for tests and full reporting of outcomes
☐	☒	Estimates of effect sizes (e.g. Cohen's *d*, Pearson's *r*), indicating how they were calculated

Our web collection on statistics for biologists contains articles on many of the points above.

Software and code

Policy information about availability of computer code

Data collection	No data collection software was used for this project.
Data analysis	No custom code was developed for this project. We used previously developed pipelines for the following analytic procedures:<br>- converting .cram to .bam (cramtools v3.0, https://stab.st-andrews.ac.uk/wiki/index.php/Cramtools)<br>- calling tandem repeat genotypes from DNA sequence reads using GangSTR v2.5.0 and MonSTR v1.1.0 (https://gymreklab.com/)<br>- kinship assignment (PLINK v1.9, https://www.cog-genomics.org/plink/)<br>- finemapping with FINEMAP v1.3.1 (http://www.christianbenner.com)<br>- assessing protein structural changes with AlphaFold v2.2.0 (https://alphafold.ebi.ac.uk)<br>- gene set enrichment with ShinyGO v0.65 (http://bioinformatics.sdstate.edu/go/#tab-1377-10) and FUMA v1.3.7 (https://fuma.ctglab.nl)<br>- generalized linear models in R (v 4.0.3) for association of tandem repeats with phenotypic data<br>- annotation of protein domains with InterPro 90.0 (https://www.ebi.ac.uk/interpro/release_notes/)<br>- annotate canonical protein sequences with UniProt (release 2022_04, https://www.uniprot.org)

For manuscripts utilizing custom algorithms or software that are central to the research but not yet described in published literature, software must be made available to editors and reviewers. We strongly encourage code deposition in a community repository (e.g. GitHub). See the Nature Portfolio guidelines for submitting code & software for further information.

Data

Policy information about availability of data

All manuscripts must include a data availability statement. This statement should provide the following information, where applicable:

- Accession codes, unique identifiers, or web links for publicly available datasets
- A description of any restrictions on data availability
- For clinical datasets or third party data, please ensure that the statement adheres to our policy

All genotype-phenotype association data generated during this study are included in this published article and its supplementary information files. The individual-level genotype data are available under restricted access to preserve participant privacy, access can be obtained by bona fide researchers through the UK Biobank Data Analysis Platform. The data for this project were accessed through approved protocol 58146.

Human research participants

Policy information about studies involving human research participants and Sex and Gender in Research.

Reporting on sex and gender	Biological sex was included as a covariate in our study design.
Population characteristics	We included age, sex, sex×age, age^2, sex×age^2, and the top 10 within-ancestry principal components as covariates.<br><br>For trio analyses:<br>The mean age of our study cohort was 42.60 years ±1.94 (male) and 43.05 years ±1.79 (female). The offspring cohort was 56.4% female.<br><br>For population-level analyses:<br>The mean age of our study cohort was 57.02 years ±8.1 (male) and 56.7 years ±7.95 (female). The population cohort was 55.0% female.<br><br>All samples had whole exome sequencing data available for analysis and all samples had phenotype data available for up to 1,844 traits.
Recruitment	There was no recruitment performed for this work. All participants were recruited at relevant UK Biobank sites.
Ethics oversight	UK Biobank has its own research ethics approval and approved research proposals need not obtain additional ethics board approval once their proposal is approved.

Note that full information on the approval of the study protocol must also be provided in the manuscript.

Field-specific reporting

Please select the one below that is the best fit for your research. If you are not sure, read the appropriate sections before making your selection.

☒ Life sciences ☐ Behavioural & social sciences ☐ Ecological, evolutionary & environmental sciences

For a reference copy of the document with all sections, see nature.com/documents/nr-reporting-summary-flat.pdf

Life sciences study design

All studies must disclose on these points even when the disclosure is negative.

Sample size	Up to 148,607 unrelated European ancestry participants
Data exclusions	Data were excluded if they did not have whole exome sequences available at the time of analysis, if they had missing phenotype information (excluded from that regression analysis only), or if their genetically predicted ancestry did not align with a European-ancestry reference population. These decisions were made to ensure high statistical power to detect TR-phenotype associations.
Replication	No replication cohort is included in this study. A subset of population TR-trait associations were re-assessed in family trios.
Randomization	Not applicable
Blinding	Not applicable

Reporting for specific materials, systems and methods

We require information from authors about some types of materials, experimental systems and methods used in many studies. Here, indicate whether each material, system or method listed is relevant to your study. If you are not sure if a list item applies to your research, read the appropriate section before selecting a response.

Materials & experimental systems

n/a	Involved in the study
☒	☐ Antibodies
☒	☐ Eukaryotic cell lines
☒	☐ Palaeontology and archaeology
☒	☐ Animals and other organisms
☒	☐ Clinical data
☒	☐ Dual use research of concern

Methods

n/a	Involved in the study
☒	☐ ChIP-seq
☒	☐ Flow cytometry
☒	☐ MRI-based neuroimaging
